# Expenditures on Mental Health Care in the Czech Republic in 2015

**DOI:** 10.1007/s11126-019-09688-3

**Published:** 2019-11-26

**Authors:** Hana M. Broulikova, Martin Dlouhy, Petr Winkler

**Affiliations:** 1 Department of Public Mental Health, National Institute of Mental Health, Topolová 748, 250 67 Klecany, Prague, Czech Republic; 2 Faculty of Informatics and Statistics, University of Economics, Prague, Czech Republic; 3 Department of Health Sciences, Faculty of Science, Vrije Universiteit Amsterdam, De Boelelaan 1105, 1081 HV Amsterdam, the Netherlands

**Keywords:** Expenditures on psychiatric care, Central and Eastern Europe, Mental health care reform, Deinstitutionalization, OECD system of health accounts

## Abstract

Expenditures on mental health care in the Czech Republic are not being published regularly, yet they are indispensable for evaluation of the ongoing reform of Czech mental health care. The main objective of this study is to estimate the size of these expenditures in 2015 and make a comparison with the last available figures from the year 2006. The estimation is based on an OECD methodology of health accounts, which structures health care expenditures according to health care functions, provider industries, and payers. The expenditures are further decomposed according to diagnoses, and inputs used in service production. The amount spent on mental health care in 2015 reached more than 13.7 billion Czech korunas (EUR 501.6 million), which represented 4.08% of the total health care expenditures. This ratio is almost identical with the 2006 share (4.14%). There are no significant changes in the relative expenditures on mental health care and in the structure of service provision. The Czech mental health care system remains largely hospital based with most of all mental health care expenditures being spent on inpatient care. Future developments in the expenditures will indicate the success of the current effort to deinstitutionalise mental health care.

**Electronic supplementary material:**

The online version of this article (10.1007/s11126-019-09688-3) contains supplementary material, which is available to authorized users.

## Introduction

The societal burden of mental health problems has so far not been met with an adequate response [1]. More than a quarter of people experience a mental health problem every year [2–5]. The societal costs of mental disorders reached 461 billion euro in Europe in 2010 [6]. Depression is now a leading cause of disability worldwide [7], and in Europe mental health problems account for 8.9% of all DALYs [8]. Yet, the average expenditures on mental disorders do not match their burden, especially in the case of Eastern Europe: While the mental health care expenditures amounted on average to 7% of the total health care budget of the old fifteen EU countries, in Eastern European countries this share reached only 3.3 % according to the WHO 2011 Mental Health Atlas.[9]. The last available estimate for the Czech Republic (2006) is 4.14% [10].

The Czech Republic is a post-communist EU country with 10.5 million inhabitants. More than 80% of total health care financing is covered by public financing [11]. This comprises of mandatory public health insurance and of public budgets with the former being the most important source of health financing in the country. Public health insurance is administered by health insurance funds, which are public organizations collecting insurance premiums and purchasing services from health providers. All Czech public health insurance funds can operate nationally and compete for members.

Mental health care is an integral part of the Czech national health system. The system annually serves more than 600 thousand people with mental health disorders [12]. Majority of patients receive outpatient care delivered by psychiatrists in private practices. There is no gate-keeping, so a patient can visit a psychiatrist without a referral from a general practitioner. Inpatient care is provided in psychiatric departments of general hospitals and in specialized psychiatric hospitals.

The mental health care reform that was launched in 2013 ought to bring about considerable changes in the services and in the expenditures [13]. The goals of the reform include improving the quality of life of people with mental illness, reducing stigmatisation, increasing the satisfaction of patients and the efficacy of psychiatric care, improving the linkage between health and social services, and humanising psychiatric care [14]. In addition, as the Czech mental health care system is still largely hospital based, one of the major aims of the reform is to shift the locus of care from large psychiatric hospitals to communities [14–17]. To enable this deinstitutionalisation, the reform introduces new mental health centres (MHCs), which should be developed for catchment areas of 100,000 inhabitants and offer community services for people with severe mental illnesses. An MHC multidisciplinary team is staffed by psychiatric nurses, social workers, psychiatrists and psychologists**.** It provides mobile case management services, crisis interventions, day care services, and out-patient psychiatric and psychological care [18]. In 2018, the first MHCs started their pilot operation, which is – similarly to other transformation activities – financed from EU funds. After the first 18 months of operation of these MHCs, public health insurance and regional social budgets are supposed to secure their sustainable financing. Consequently, the OECD accounts will allow us to assess whether the effort to deinstitutionalise patients is reflected in the change of the structure of financed services. The shifts in expenditures from inpatient to outpatient care constitute an important indicator of the reform’s success which is why it is important to closely monitor this indicator now as well as in the future.

The aim of this article is to provide up to date estimates of the mental health expenditures in the Czech Republic and to analyse their development. Consistently with the last estimates from 2006, we aimed to use the OECD methodology of health accounts that has been developed to provide a consistent guidance on health expenditures calculation [19, 20]. This methodology provides a standardised framework allowing to sort expenditures according to different perspectives and to answer the questions who pays for mental health services, who the providers are and what services they deliver. Consequently, the resulting figures are suitable for international comparison. Although the Czech Statistical Office annually publishes health accounts based on the OECD methodology, the reported expenditures cannot be disaggregated according to diagnostic groups and, thus, information about *mental* health expenditures cannot be retrieved. Our study combines multiple data sources to fill this gap. In addition to the standard OECD perspectives, we estimate expenditures according to diagnoses and according to inputs used in service production.

## Methods

We adhered to the OECD methodology on health accounts SHA 1.0 [20] and its revisions SHA 2011 [19]. This methodology constructs the accounts from three different perspectives: health care functions (ICHA–HC, Table 1), provider industries (ICHA–HP, Table 2), and payers (ICHA–HF, Table 3). Each of the perspectives divides health care expenditures into individual chapters according to a different criterion. The health care function account attributes expenditures for example to services of curative care, and rehabilitative care. The provider industry perspective sorts expenditures according to the type of facility within which they were incurred, including hospitals, or providers of ambulatory care. The health care function account as well as the provider industry account include also chapters on medical goods provision, prevention, and health care administration. The perspective of payers categorizes expenditures according to who incurred them into the chapters of government and compulsory health insurance financing schemes, voluntary financing schemes, and household out-of-pocket payment.

**Table 1:** Mental health expenditures by the OECD ICHA-HC classification of health-care services/functions, Czech Republic, 2015

Health care function	Mental health expenditures 2015, millions CZK (EUR)	Share of function, 2015 (2006)
HC.1 Curative care	10,272 (376.5)	75.1% (71.7%)
HC.1.1 Inpatient curative care	8361 (306.5)	61.1% (56.3%)
HC.1.2 Day curative care	0	0% (0%)
HC.1.3 Outpatient curative care	1910 (70)	14% (15.3%)
HC.1.4 Home-based curative care	0	0% (0%)
HC.2 Rehabilitative care	59 (2.2)	0.4% (0.4%)
HC.2.1 Inpatient rehabilitative care	59 (2.2)	0.4% (0.4%)
HC.2.2 Day rehabilitative care	0	0% (0%)
HC.2.3 Outpatient rehabilitative care	0	0% (0%)
HC.2.4 Home-based rehabilitative care	0	0% (0%)
HC.3 Long-term care (health)	0	0% (0%)
HC.4 Ancillary services (non-specified by function)	286 (10.5)	2.1% (0%)
HC.4.1 Laboratory services	ND	
HC.4.2 Imaging services	ND	
HC.4.3 Patient transportation	286 (10.5)	2.1% (0%)
HC.5 Medical goods (non-specified by function)	2713 (99.4)	19.8% (25.9%)
HC.5.1 Pharmaceuticals and other medical non-durable goods	2713 (99.4)	19.8% (25.9%)
HC.5.2 Therapeutic appliances and other medical goods	0	0% (0%)
HC.6 Preventive care	0	0% (0%)
HC.7 Governance, and health system and financing administration	356 (13)	2.6% (2.1%)
HC.7.1 Governance and health system administration	ND	
HC.7.2 Administration of health financing	ND	
Total	13,685 (501.6)	100%

**Table 2:** Mental health expenditures by the OECD ICHA-HP classification of health providers, Czech Republic, 2015

Health care provider	Mental health expenditures 2015, millions CZK (EUR)	Share of provider, 2015 (2006)
HP.1 Hospitals	8608 (315.5)	62.9% (59%)
HP.1.1 General hospitals	926 (33.9)	6.8% (6.6%)
HP.1.2 Mental health hospitals	7623 (279.4)	55.7% (52.4%)
HP.1.3 Specialised hospitals (other than mental health hospitals)	59 (2.2)	0.4% (0:4%)
HP.2 Residential long-term care facilities	0	0% (0%)
HP.3 Providers of ambulatory health care	1723 (63.1)	12.6% (12.6%)
HP.3.1 Medical practices	1723 (63.1)	12.6% (12.6%)
HP.3.2 Dental practice	0	0% (0%)
HP.3.3 Other health care practitioners	ND	
HP.3.4 Ambulatory health care centres	ND	
HP.3.5 Providers of home health care services	ND	
HP.4 Providers of ancillary services	286 (10.5)	2.1% (0%)
HP.4.1 Providers of patient transportation and emergency rescue	286 (10.5)	2.1% (0%)
HP.4.2 Medical and diagnostic laboratories	ND	
HP.4.9 Other providers of ancillary services	ND	
HP.5 Retailers and other providers of medical goods	2713 (99.4)	19.8% (25.9%)
HP.5.1 Pharmacies	ND	
HP.5.2 Retail sellers and other suppliers of durable medical goods and medical appliances	ND	
HP.5.9 All other miscellaneous sellers and other suppliers of pharmaceuticals and medical goods	ND	
HP.6 Providers of preventive care	0	0% (0%)
HP.7 Providers of health care system administration and financing	356 (13)	2.6% (2.1%)
HP.7.1 Government health administration agencies	ND	
HP.7.2 Social health insurance agencies	ND	
HP.7.3 Private health insurance administration agencies	ND	
HP.7.9 Other administration agencies	ND	
HP.8 Rest of economy	0	0% (0%)
HP.9 Rest of the world	0	0% (0%)
Total	13,685 (501.6)	100%

**Table 3:** Mental health expenditures by the OECD ICHA-HF classification of health-care financing, Czech Republic, 2015

Health-care financing	Mental health expenditures 2015, millions CZK (EUR)	Share of financing budget, 2015 (2006)
HF.1 Government schemes and compulsory contributory health care financing schemes	11,582 (424.5)	84.6% (88%)
HF.1.1 Government schemes	2501 (91.7)	18.3% (10.3%)
HF.1.1.1 Central government schemes	2124 (77.8)	15.5% (6.5%)
HF.1.1.2 State/regional/local government schemes	378 (13.8)	2.8% (3.8%)
HF.1.2 Compulsory contributory health insurance schemes	9080 (332.8)	66.4% (77.7%)
HF.1.3 Compulsory Medical Saving Accounts (CMSA)	0	0% (0%)
HF.2 Voluntary health care payment schemes	361 (13.2)	2.6% (0.5%)
HF.2.1 Voluntary health insurance schemes	18 (0.7)	0.1% (0%)
HF.2.2 NPISH financing schemes	307 (11.2)	2.2% (0%)
HF.2.3 Enterprise financing schemes	35 (1.3)	0.3% (0.5%)
HF.3 Household out-of-pocket payment	1743 (63.9)	12.7% (11.5%)
Total	13,685 (501.6)	100%

Several remarks are needed as far as the construction of our accounts is concerned. The methodology sometimes asks for a higher degree of disaggregation than we were able to achieve; when this is the case, the table shows not disaggregated (ND) for the respective subchapter. We also had to address the changes brought by the revised version of the methodology SHA 2011. First, the SHA 2011 renames some chapters and subchapters, and slightly changes the structure of the accounts. We reassigned the 2006 expenditures to facilitate comparisons. Second, paying greater attention to social services provided to patients, SHA 2011 introduces a new chapter long-term social care (HCR.1). This expands the calculated size of the total health care budget. To allow comparability with the 2006 figure, we report both the 2015 share of mental health expenditures in the total health expenditure including the new chapter (SHA 2011) as well as the share in the total expenditure that excludes it (SHA 1.0).

In addition to the OECD methodology, we further classify expenditures according to groups of related diagnoses (Table 4). These groups are based on the internationally recognized 10th revision of the International Statistical Classification of Diseases and Related Health Problems (ICD-10) [21]. Lastly, we also consider the perspective of inputs entering production of health care services (Table 5). The complete methodological procedure and results are available in Online Resource 1.

**Table 4:** Mental health expenditures by the ICD-10 diagnostic groups and by health Provider, Czech Republic, 2015

Diagnostic structure	HP.3.1 Medical practices 2015, CZK (EUR)	HP.5 Retailers and other providers of medical goods 2015, CZK (EUR)	HP.1.1 General hospitals 2015, CZK (EUR)	HP.1.2 Mental health hospitals 2015, CZK (EUR)	Total diagnosis 2015, CZK (EUR)	Total diagnosis in %, 2015 (2006)
F00-F09 Organic, including symptomatic, mental disorders	269 (9.9)	321 (11.8)	121 (4.4)	1595 (58.5)	2306 (84.5)	17.76%
F10 Mental and behavioural disorders due to use of alcohol	60 (2.2)	37 (1.4)	97 (3.5)	1015 (37.2)	1209 (44.3)	9.31%
F11-F19 Mental and behavioural disorders due to use of other psychoactive substances	31 (1.1)	26 (1.0)	61 (2.2)	454 (16.6)	571 (20.9)	4.40%
F20-F29 Schizophrenia, schizotypal and delusional disorders	186 (6.8)	853 (31.3)	142 (5.2)	2687 (98.5)	3868 (141.8)	29.78%
F30-F39 Mood (affective) disorders	190 (7.0)	506 (18.6)	112 (4.1)	373 (13.7)	1182 (43.3)	9.10%
F40-F48; F50-F59 Neurotic, stress-related and somatoform disorders	493 (18.1)	601 (22.0)	234 (8.6)	429 (15.7)	1758 (64.4)	13.54%
F60-F63; F68-F69 Disorders of adult personality and behaviour	51 (1.9)	66 (2.4)	36 (1.3)	266 (9.8)	419 (15.4)	3.23%
F64-F66 Sexual disorders	4 (0.1)	6 (0.2)	8 (0.3)	133 (4.9)	151 (5.5)	1.16%
F70-F79 Mental retardation	173 (6.3)	145 (5.3)	22 (0.8)	455 (16.7)	795 (29.1)	6.12%
F80-F98 Disorders of psychological development and behavioural and emotional disorders	257 (9.4)	151 (5.5)	91 (3.3)	213 (7.8)	712 (26.1)	5.48%
F99 Unspecified mental disorder	10 (0.4)	1 (0.1)	2 (0.1)	2 (0.1)	15 (0.5)	0.11%
Total	1723 (63.1)	2713 (99.4)	926 (33.9)	7623 (279.4)	12,985 (475.9)	100%

**Table 5:** Mental health expenditures by cost category, general and mental health hospitals only, Czech Republic, 2015

Category	General hospital in %, 2015 (2006)	Psychiatric hospital in %, 2015 (2006)	General hospital expenditure 2015, millions CZK (EUR)	Psychiatric hospital expenditure 2015, millions CZK (EUR)	Category total 2015, millions CZK (EUR)
Personal Cost	46.3% (43.7%)	67.2% (63.7%)	429 (15.7)	5123 (187.8)	5551 (203.5)
Drugs	13.7% (8.3%)	3.3% (4.1%)	127 (4.7)	252 (9.2)	378 (13.9)
Special medical materials	12.8% (13.9%)	1.6% (1.9%)	119 (4.3)	122 (4.5)	241 (8.8)
Blood	1.1% (1.2%)	0% (0%)	10 (0.4)	0	10 (0.4)
Food	0.8% (1.1%)	4.7% (4.9%)	7 (0.3)	358 (13.1)	366 (13.4)
Energy	3.1% (3.1%)	5.9% (6.3%)	29 (1.1)	450 (16.5)	478 (17.5)
Services	7.2% (8%)	7.3% (7.7%)	67 (2.4)	556 (20.4)	623 (22.8)
Depreciation	4.6% (5.4%)	3.3% (3.1%)	43 (1.6)	252 (9.2)	294 (10.8)
Other	10.4% (15.2%)	6.6% (8.3%)	96 (3.5)	503 (18.4)	599 (22)
Total	100%	100%	926 (33.9)	7623 (279.4)	8541 (313.1)

### Data

The main data sources are public reports collected from the General Health Insurance Fund (GHIF), the Czech Statistical Office (CSO), and The Institute of Health Information and Statistics of the Czech Republic (IHIS). These resources were complemented by unpublished information from the Ministry of Health of the Czech Republic and GHIF. GHIF is the largest health insurer in the Czech Republic. Two thirds of the population are enrolled with this insurer, which provides a certain guarantee of representativeness [22]. The insurer annually publishes a comprehensive yearbook [23] with a detailed description of enrolees, collected premiums, services provided by contracted health facilities, and related expenditures. This document specifies total expenditures, expenditures on psychopharmaceuticals, and expenditures on rehabilitative spas. The GHIF further provided unpublished financial data on ambulatory psychiatric care, psychiatric hospitals, and psychiatric departments in general hospitals as well as the relative resource consumption by each diagnostic group. The CSO annually publishes accounts based on the OECD methodology for the entire national system of health care [11]. For our analysis, we use the information about total health care expenditures and expenditures on health administration contained in these accounts. In 2013, IHIS reported the structure of costs for selected types of health care establishments that serves as a basis for our input category perspective [24]. The results are converted from Czech korunas (CZK) to euro (EUR) with the annual average of the daily nominal exchange rate in 2015: EUR 1 = CZK 27.283 [25].

Mental health expenditures are defined as health expenditures on services for patients with primary or first-listed diagnoses from Chapter V, Mental and Behavioural Disorders (F00-F99), of the Tenth Revision of International Classification of Diseases (ICD-10). By this definition we exclude expenditures on somatic illnesses that can be partially a consequence of mental health conditions (for example cirrhosis of liver as a consequence of alcohol addiction). The study further excludes disability pensions, sickness benefits, and also services for the mentally ill that are considered as social services in the Czech context. Such types of expenditures are financed from social care budgets, mainly by central and local governments. Unfortunately, there is no reliable data source that would allow us to identify the share of the social care budget allocated to mental health issues. Due to a lack of reliable data, the study also excludes services for the mentally ill provided by general practitioners.

### Health Care Function Perspective (ICHA-HC)

The health care function perspective allocates expenditures to the following chapters and subchapters that are applicable in the context of the Czech Republic: curative care (chapter HC.1, subchapters HC.1.1 inpatient curative care in psychiatric and general hospitals and HC.1.3 outpatient curative care), rehabilitative care (HC.2, subchapter HC.2.1 inpatient rehabilitative care), ancillary services (HC.4, subchapter HC.4.3 patient transportation), medical goods (HC.5, subchapter HC.5.1 pharmaceuticals and other medical non-durable goods), and governance and health system financing and administration (HC.7, data not disaggregated into subchapters). Rehabilitative care refers in the Czech context to health spa. Patient transportation includes emergency cases only. Medical goods denote outpatient pharmaceuticals while inpatient pharmaceuticals are a part of inpatient expenditures.

First, we calculated the shares of particular functions on the total yearly expenditures of GHIF (from both published [23] and unpublished sources). The sum of these shares gives a relative proportion of mental health care expenditures to total health care expenditures. Second, assuming that for other insurers and sources of financing the shares are equivalent, we subsequently applied them to the total national health care expenditures reported by the CSO [11] to get mental health expenditures for the whole system. Expenditures on preventive care (chapter HC.6 of ICHA–HC), long-term social care (chapter HCR.1), health promotion (chapter HCR.2), investments, education, and research and development (chapters R.1, R.2, R.3) were excluded from the amount of total health care expenditures. The reason is that most of these chapters are complementary to health care expenditures rather than constituting their organic part. Moreover, we expect a large heterogeneity in spending across particular health care fields within these chapters. Consequently, applying the assumption that expenditures on these chapters correspond to the fraction of the total budget of GHIF consumed by mental health care would mislead the results. On the other hand, we consider reasonable to apply this assumption to mental-health care administration (chapter HC.7) as there is no compelling reason why mental-health care administration should be differently demanding than administration in other health care fields.

### Provider Industry Perspective (ICHA-HP)

To categorize expenditures according to provider industries, figures on health care functions were clustered together according to institutional settings in which care is provided. The most relevant chapters are hospitals (HP.1) and providers of ambulatory health care (HP.3). Hospitals are further subdivided into general hospitals (HP.1.1), psychiatric hospitals (HP.1.2), and health spas (coded as specialized hospitals HP.1.3). The ambulatory care provided in hospitals is allocated to subchapter HP.1.1 and ambulatory care provided by independent medical practices is allocated to subchapter HP.3.1. The chapters representing providers of medical goods (HP.5) and health administration (HP.7) display the same figures as the corresponding chapters under the health care function perspective.

### Payer Perspective (ICHA-HF)

To offer the payer perspective, we start from the CSO classification of national health expenditures by the type of the financing entity. The applicable chapters are government schemes and compulsory contributory health care financing schemes (HF.1, subchapters HF.1.1.1/2 government schemes further divided to the central and local government schemes, and HF.1.2 compulsory contributory health insurance schemes), voluntary health care payment schemes (HF.2, subchapters voluntary health insurance schemes HF.2.1, non-profit financing schemes HF.2.2, and enterprise financing schemes HP2.3), and household out-of-pocket payment (HF.3).

We assume that the shares of different budgeting segments are the same for the mental health care accounts as for the general health care accounts. We calculate the share of each budgeting segment and apply it to the national expenditures on mental health care.

### Diagnosis Perspective

An unpublished data from GHIF shows how its reimbursements to providers of mental health care are divided among different diagnostic groups. We consider separately outpatient medical practices (HP.3.1), psychiatric departments of general hospitals (HP.1.1), psychiatric hospitals (HP.1.2) and providers of medical goods (HP.5). To extrapolate the information to the whole system, we multiply the estimates of expenditures on a given provider industry from the table ICHA-HP with the share that each diagnostic group consumes according to GHIF.

### Input Costs Perspective

To divide expenditures according to input categories such as labour or material, we use shares of inputs published by IHIS in 2013, which cover psychiatric as well as general hospitals. We assume that shares of input costs at psychiatric departments of general hospitals correspond to the shares of input costs at general hospitals as a whole. Expenditures on inputs for psychiatric hospitals and departments are then calculated by division of expenditures per a type of provider in 2015 according to the shares published by IHIS.

## Results

National mental health care expenditures reached 13.7 billion CZK (EUR 501.6 million), which is 1297 CZK (EUR 48) per capita in the Czech Republic in 2015. Relatively, mental health care expenditures represent 4.08% of the total health care budget according to the original guidelines SHA 1.0 [20]. The inclusion of a chapter on long run social care (HCR.1) recognized by the revised version SHA 2011 [19] into the total budget further decreases the estimate of mental health share to 3.87%.

Three quarters of the mental health care budget is spent on services of curative care. These expenditures are mainly driven by inpatient care, costing 8.4 billion (EUR 306.5 million, 61.1% of the entire budget). To compare, outpatient care cost 1.9 billion (EUR 70 million, 14%). Hospitals consumed 8.6 billion (EUR 315.5 million, 62.9%). Specifically, 7.6 billion (EUR 279.4 million, 55.7%) was spent on psychiatric hospitals, with the rest of the chapter being allocated to psychiatric departments of general hospitals and mental health spas. The largest part (30%) of the budget was allocated to schizophrenia, schizotypal and delusional disorders (F20-F29). The share of public financing reached 84.6% in 2015 [11]. Public budgets and public health insurance spent 11.6 billion (EUR 424.5 million) on mental health care. Of this amount, 9.1 billion (EUR 332.8 million) was paid by public health insurers. The most expensive input used in the production of mental health care was labour (65%, CZK 5.6 billion, EUR 203.5 million). Tables 1, 2, 3, 4 and 5 report complete results categorized from the perspective of health care functions (Table 1), provider industries (Table 2), source of financing (Table 3), diagnostic groups (Table 4) and input cost categories (Table 5).

## Discussion

While the period between 2001 and 2006 marked an increase in the share of mental health care on the total health care expenditures from 3.54% [10] to 4.14% [26], our results suggest that this share remained roughly constant during the last decade, reaching 4.08% in 2015. This means that the Czech share of mental health expenditures amounts only to two thirds of the median value 6.3% observed in the European WHO region [9, 27], and two fifths of what is recommended [1]. Such a low proportion of health budget spent on mental health is clearly a sign of structural discrimination and a failure to allocate resources that would correspond to the overall societal burden caused by mental disorders [1].

The structure of expenditures has changed only slightly between 2006 and 2015. The share of expenditures on curative care (HC.1) increased from 71.7% to 75.1% mainly due to a growth in resources allocated to inpatient curative care (HC.1.1, from 56.3% to 61.1%) in psychiatric hospitals (HP.1.2 from 52.4 to 55.7%). This development is mirrored in a relative decrease of expenditures on outpatient pharmaceuticals (HC.5.1), which dropped from 25.9% to 19.8% of the total expenditures on mental health care.

We further observed a slight decrease in the share of expenditures paid from public budgets (HF.1) from 88% in 2006 to 84.6% in 2015. Within this chapter, the contribution of compulsory health insurance schemes (HF.1.2) dropped from 77.7% to 66.4%. In contrast, the share of central and local governments increased from 10.3% to 18.3%. In addition, more resources were paid through voluntary health care payment schemes (HF.2, increase from 0.5% to 2.6%). The main change within this chapter pertains to the jump from 0 to 2.2% in the case of non-profit organizations (HF.2.2). However, the question is whether this change reflects actual differences in financing or just a new statistical awareness of this type of expenditures. Interestingly, private out-of-pocket expenditures remain stable (11.5% vs. 12.7%).

Our estimates on resources consumed by different diagnostic groups correct the figures from 2006, which were based on a strong assumption that – within each industry – care is equally expensive for every patient regardless of his or her psychiatric diagnosis. Consequently, in 2006, the diagnostic group deemed as most costly consisted of neurotic, stress related and somatoform disorders (F40-F48; F50-F59) due to a large number of patients suffering from these disorders. Although the shares of patients remained similar (see Online Resource 1, sheet *Patients*), in 2015, the most expensive group of diagnoses included schizophrenia, schizotypal and delusional disorders (F20-F29) consuming 30% of the budget. To illustrate this disproportionality in respect to a particular industry, whereas the said disorders accounted only for 19% of patients in psychiatric hospitals, they consumed 35% of the hospitals’ budget. Another recent study [28] moreover shows that the costs of community care for these disorders – and for other non-affective psychoses – are significantly lower than the costs of their inpatient care (about 350 thousand CZK per patient) while being similarly effective. Schizophrenia, schizotypal and delusional disorders thus appear to be apt candidates for deinstitutionalization of the care [10, 29]. The success of the reform effort to achieve deinstitutionalization, concerning this as well as other diagnostic groups, will be indicated by future changes in the expenditures and mainly in their structure.

The strength of this study is that we use official data provided by reliable institutions to construct mental health care accounts according to an internationally recognized methodology. Moreover, the unavoidable assumptions used for calculations are consistent with the similar study conducted in 2006, which enables time comparison.

The results might nevertheless be influenced by reporting practices of the CSO. Its adoption of the new OECD methodology led to an immediate increase in some of the account chapters, especially in regard to long term care; the total and administrative expenditures, which we use in our calculations, might hence be distorted. Further, it is not clear to what extent the accounts reflect expenditures on some mental health services on the boundary of social and health care such as the community centres, which are paid for by the Ministry of Labour and Social Affairs instead of by public health insurers. Since we work with the composition of services financed by the GHIF as one of these insurers, our calculations fail to assign the community centre expenditures (to the extent to which the CSO actually does include them in the figures that it reports) to the category of outpatient care. Instead, our methodology divides these expenditures among all categories of services according to their shares calculated from the GHIF data. As a result, the category of outpatient care could be biased downwards. Nevertheless, we believe that the magnitude of error is modest because community services are still provided on a rather small scale in the Czech Republic [14]. Another distortion in the expenditures structure was caused by an unavailability of information on the emergency service expenditures (chapters HC.4.3 and HP.4.1 respectively) for the year 2006. As a result of these comparability issues, the subtle changes in the structure of expenditures that we observe should not be seen as conclusive manifestations of actual expenditure trends.

## Conclusion

This study estimates expenditures on mental health care in the Czech Republic in 2015. The results suggest that the share of these expenditures on the total health care budget had remained constant over the previous ten years, staying well below the European average. Developments in mental health expenditures will serve as an important indicator for evaluation of the current effort to deinstitutionalise mental health care. An important task for future research is to investigate the part of these expenditures that is incurred by social care budgets, mainly as regards community mental health care.

## Electronic supplementary material

ESM 1 (XLSX 50 kb)
